# [^89^Zr]Zr-PSMA-617 PET/CT in biochemical recurrence of prostate cancer: first clinical experience from a pilot study including biodistribution and dose estimates

**DOI:** 10.1007/s00259-022-05925-3

**Published:** 2022-08-05

**Authors:** Florian Rosar, Andrea Schaefer-Schuler, Mark Bartholomä, Stephan Maus, Sven Petto, Caroline Burgard, Bastiaan M. Privé, Gerben M. Franssen, Yvonne H. W. Derks, James Nagarajah, Fadi Khreish, Samer Ezziddin

**Affiliations:** 1 Department of Nuclear Medicine, Saarland University – Medical Center, Kirrberger Str. 100, Geb. 50, 66421 Homburg, Germany; 2 Department of Medical Imaging, Nuclear Medicine, Radboud University Medical Center, Nijmegen, The Netherlands

**Keywords:** Prostate cancer, PSMA, PET/CT, Zirconium-89, Biochemical recurrence

## Abstract

**Purpose:**

Prostate-specific membrane antigen (PSMA)-targeted PET/CT has become increasingly important in the management of prostate cancer, especially in localization of biochemical recurrence (BCR). PSMA-targeted PET/CT imaging with long-lived radionuclides as ^89^Zr (T_1/2_ = 78.4 h) may improve diagnostics by allowing data acquisition on later time points. In this study, we present our first clinical experience including preliminary biodistribution and dosimetry data of [^89^Zr]Zr-PSMA-617 PET/CT in patients with BCR of prostate cancer.

**Methods:**

Seven patients with BCR of prostate cancer who revealed no (*n* = 4) or undetermined (*n* = 3) findings on [^68^Ga]Ga-PSMA-11 PET/CT imaging were referred to [^89^Zr]Zr-PSMA-617 PET/CT. PET/CT imaging was performed 1 h, 24 h, 48 h, and 72 h post injection (p.i.) of 111 ± 11 MBq [^89^Zr]Zr-PSMA-617 (mean ± standard deviation). Normal organ distribution and dosimetry were determined. Lesions visually considered as suggestive of prostate cancer were quantitatively analyzed.

**Results:**

Intense physiological uptake was observed in the salivary and lacrimal glands, liver, spleen, kidneys, intestine and urinary tract. The parotid gland received the highest absorbed dose (0.601 ± 0.185 mGy/MBq), followed by the kidneys (0.517 ± 0.125 mGy/MBq). The estimated overall effective dose for the administration of 111 MBq was 10.1 mSv (0.0913 ± 0.0118 mSv/MBq). In 6 patients, and in particular in 3 of 4 patients with negative [^68^Ga]Ga-PSMA-11 PET/CT, at least one prostate cancer lesion was detected in [^89^Zr]Zr-PSMA-617 PET/CT imaging at later time points. The majority of tumor lesions were first visible at 24 h p.i. with continuously increasing tumor-to-background ratio over time. All tumor lesions were detectable at 48 h and 72 h p.i.

**Conclusion:**

[^89^Zr]Zr-PSMA-617 PET/CT imaging is a promising new diagnostic tool with acceptable radiation exposure for patients with prostate cancer especially when [^68^Ga]Ga-PSMA-11 PET/CT imaging fails detecting recurrent disease. The long half-life of ^89^Zr enables late time point imaging (up to 72 h in our study) with increased tracer uptake in tumor lesions and higher tumor-to-background ratios allowing identification of lesions non-visible on [^68^Ga]Ga-PSMA-11 PET/CT imaging.

## Introduction

Over the past decade, prostate-specific membrane antigen (PSMA)-targeted positron emission tomography (PET)/computed tomography (CT) has revolutionized imaging of patients with prostate cancer [1]. PSMA is a transmembrane glycoprotein, which is overexpressed on the cell surface of prostate carcinoma cells [2], providing an ideal and specific target for imaging and therapy [3, 4]. PSMA-targeted PET/CT has become increasingly important in the management of prostate cancer for initial staging, localization of biochemical recurrence, and screening or monitoring of PSMA-targeted radioligand therapy [5–8]. In recent years, various PSMA-ligands have been developed, of which ^68^Ga-labeled PSMA-11 and ^18^F-labeled DCFPyL or PSMA-1007 have become widely used for PET/CT imaging in clinical practice, and ^177^Lu-labeled PSMA-617 for radioligand therapy [9, 10].

[^68^Ga]Ga-PSMA-11 has shown a high sensitivity for tumor localization in the setting of biochemical recurrence (BCR) of prostate cancer in various recent prospective studies; however, there remains a non-negligible number of patients with BCR and negative [^68^Ga]Ga-PSMA-11 PET/CT, particularly in patients with low PSA levels [11–14]. Due to its short half-life of 68 min, ^68^Ga does not allow late image acquisition (e.g., on the next day after injection); therefore, imaging is usually performed 1 h after injection [15]. Similar applies to ^18^F-labeled PSMA-ligands with ^18^F offering a moderately longer half-life of 110 min. However, [^18^F]F-PSMA-1007 offers a possible advantage for the detection of local recurrences due to a lower urinary excretion [16]. But also for [^18^F]F-PSMA-1007, a certain number of patients with BCR and negative PSMA PET/CT have been observed [17].

PET/CT imaging with PSMA ligands labeled with long-lived radionuclides may increase sensitivity of PSMA PET/CT by allowing longer clearance from non-target organs and therefore resulting in increased target-to-background ratios. In addition, late acquisitions could also increase specificity by confirming or negating undetermined findings by observing uptake over time. In this context, the use of ^89^Zr may be of interest [18]. ^89^Zr is a positron emitter (branching fraction 23%) with a half-life of T_1/2_ = 78.4 h and a mean positron energy of 0.395 MeV, which is frequently used for antibody imaging [19–21]. Due to chemical reasons, ^89^Zr cannot be complexed by PSMA-11, but binds to the commonly used bifunctional chelators DOTA and DOTAGA, thus allowing the radiolabeling of PSMA-617 and PSMA I&T [22, 23]. Recently, we described the preclinical characterization of [^89^Zr]Zr-PSMA-617 and [^89^Zr]Zr-PSMA I&T including biodistribution studies in tumor bearing mice [23]. Here, we present our first clinical experience including biodistribution and preliminary dosimetry estimates of [^89^Zr]Zr-PSMA-617 PET/CT in patients with BCR of prostate cancer.

## Methods

### Patients and ethics

[^89^Zr]Zr-PSMA-617 PET/CT imaging was performed in *n* = 7 consecutive patients due to BCR of prostate cancer who revealed no or undetermined findings on [^68^Ga]Ga-PSMA-11 PET/CT. In 4 patients, [^68^Ga]Ga-PSMA-11 did not reveal suspicious findings (negative [^68^Ga]Ga-PSMA-11 PET/CT). Three patients had undetermined findings on [^68^Ga]Ga-PSMA-11 PET/CT with no definite assignment to pathological or physiological uptake, e.g., faintly accumulating or unusually located tracer uptake. The time interval between both, [^89^Zr]Zr-PSMA-617 and [^68^Ga]Ga-PSMA-11 PET/CT, was 31 ± 18 days (range 5–49 days), with no treatment performed in between. Prostate-specific antigen (PSA) serum level at time of imaging ranged from 0.43 to 1.92 ng/ml. All patients were initially treated with radical prostatectomy (RP). Initial Gleason score ranged from 7a to 9. Four patients underwent salvage treatments such as androgen-deprivation therapy (ADT), lymphadenectomy (LA) or radiation therapy (RT). Detailed patient characteristics including age, Gleason score, primary therapy, salvage therapies, time from initial diagnosis (ID) of prostate cancer, PSA and PSA doubling time (DT) are presented in Table 1. [^89^Zr]Zr-PSMA-617 PET/CT imaging was performed on a compassionate use basis under the German Pharmaceutical Act §13 (2b). The medical indication for the examination and the labeling of the tracer were under the direct responsibility of the applying physician. Patients gave their written consent after being thoroughly informed about the general risks of both radiation exposure and application of a novel PET tracer including possible adverse effects of, e.g., therapeutic PSMA tracers. Furthermore, all patients agreed to the publication of the resulting data in accordance with the Declaration of Helsinki.

**Table 1:** Patient characteristics

Pt. no	Age (years)	Gleason score	Primary therapy	Salvage therapies	Current setting	Time from ID (years)	PSA (ng/ml)	PSA DT (months)	Findings on [^68^Ga]Ga-PSMA-11 PET/CT	Findings on [^89^Zr]Zr-PSMA-617 PET/CT
1	66	7a	RP	-	BCR	4	1.92	6	No suspicious findings	Local recurrence
2	64	8	RP	ADT, LA, RT	BCR	3	1.75	> 12	1 × LNM + undetermined finding (suspicion of right iliac LNM)	3 × LNM (no right iliac LNM)
3	69	7b	RP	ADT, RT	BCR	10	0.66	9	No suspicious findings	No suspicious findings
4	74	7b	RP	LA, RT	BCR	8	1.77	4	Undetermined finding (suspicion of peritoneal metastasis)	Peritoneal metastasis + 4 × LNM
5	70	8	RP	ADT, RT	BCR	4	0.63	4	No suspicious findings	1 × LNM
6	72	8	RP	-	BCR	19	0.43	> 12	Undetermined finding (suspicion of local recurrence in seminal vesicle bed)	Local recurrence (in seminal vesicle bed)
7	67	9	RP	-	BCR	1	0.68	> 12	No suspicious findings	Local recurrence + 2 × LNM

*ADT*, androgen-deprivation therapy; *BCR*, biochemical recurrence; *DT*, doubling time; *ID*, initial diagnosis of prostate cancer; *LA*, lymphadenectomy; *LNM*, lymph node metastasis; *PSA*, prostate-specific antigen; *RP*, radical prostatectomy; *RT*, radiation therapy

### Synthesis and quality control

The radiolabeling of PSMA-617 with ^89^Zr was based on our previously published procedure with further optimizations [23]. Briefly, [^89^Zr]Zr-oxalate (PerkinElmer, Groningen, The Netherlands) was transformed into [^89^Zr]ZrCl_4_ using a QMA cartridge (Waters, Milford, USA), which was activated by each 10 mL of acetonitrile, saline, 1 M hydrochloric acid, and deionized water. [^89^Zr]Zr-oxalate was then loaded onto the cartridge followed by a washing step with 60 mL of deionized water. The activity was recovered from the QMA cartridge by fractionated elution with two fractions of 700 µL and 800 µL of 0.1 M hydrochloric acid. The latter fraction contained ~ 95% of the initial activity and was used for radiolabeling. To this fraction, 1.5 µg (1.44 nmol) of PSMA-617 per MBq [^89^Zr]ZrCl_4_ in 50 µL water were added followed by the addition of 800 µL MES buffer (0.5 M, pH 5.5). The reaction mixture was then heated at 95 °C for 30 min. After cooling to room temperature, the mixture was loaded onto a C_18_ Sep Pak cartridge (Waters, Milford, USA), which was pre-equilibrated with each 10 mL of ethanol and water for injection. The cartridge was washed with 5 mL of water for injection and the activity eluted with 2 mL 50% ethanol (v/v) and 8 mL of saline. The product was finally passed through a 0.22-µm filter for sterilization. This procedure is reliable for up to 800 MBq of initial ^89^Zr activity providing the final product [^89^Zr]Zr-PSMA-617 in radiochemical yields of 70 ± 5% and radiochemical purities of > 98%. Quality control of the final product was performed according to current Good Manufacturing Practice (cGMP) guidelines checking for pH, clarity and color, radiochemical purity (HPLC and iTLC), chemical purity (HPLC), radionuclidic purity, endotoxin content, and sterility.

### PET/CT imaging

Each patient underwent PET/CT imaging scans at 4 time points: 1 h, 24 h, 48 h, and 72 h post intravenous injection (p.i.) of 111 ± 11 MBq (mean ± standard deviation, range 97–129 MBq) [^89^Zr]Zr-PSMA-617. All PET/CT imaging was performed on a Biograph mCT 40 scanner (Siemens Medical Solutions, Knoxville, TN, USA) comprising whole-body PET/CT scans extending from vertex to mid-femur in 3D mode. PET acquisition time duration was 4 min per bed position on the initial day of injection and extended up to 10 min on the last day. CT data were acquired in low-dose technique using an X-ray tube voltage of 120 keV and a modulation of the tube current (CARE Dose4D, Siemens Erlangen; maximal tube current 30 mA) followed by reconstruction with a soft tissue reconstruction kernel (B31f) to a slice thickness of 5 mm (increment 2–4 mm). PET emission data was corrected for decay, randoms, and scatter. PET image reconstruction was performed applying an iterative 3-dimensional ordered-subset expectation maximization (OSEM) algorithm (3 iterations; 24 subsets) with gaussian filtering to a transaxial resolution of 5 mm at full width half maximum (FWHM). The matrix and the pixel size were 200 × 200 and 3.0 mm, respectively. Attenuation correction was performed using the low-dose CT data.

### Adverse events

Any adverse event was recorded during and after examination. Vital parameters as heart rate, blood pressure, body temperature, and oxygen saturation were closely monitored. Within a time interval of 4 weeks after [^89^Zr]Zr-PSMA-617 PET/CT, patients were additionally interviewed about potential side effects.

### Biodistribution and tumor uptake

The biodistribution of [^89^Zr]Zr-PSMA-617 was quantified by analyzing the standard uptake values (SUV) SUV_max_, SUV_peak_ and SUV_mean_ at 1 h, 24 h, 48 h, and 72 h p.i.. Considering normal-organs, elliptical volumes of interest (VOI) were manually drawn enclosing regions of relatively homogenous uptake. Activity estimation was performed within the VOI applying a 40% or, in case of faintly accumulating organs, a 20% threshold using the SyngoVia software (Enterprise VB 60, Siemens, Erlangen, Germany). The following organs were included in this evaluation: the brain, salivary, and lacrimal glands, nasal mucosa, lung, liver, spleen, small and large intestine, and the kidneys. With regard to the large intestine, SUV was determined at the descending colon. Blood pool and background were evaluated in the descending aorta and the gluteal muscle, respectively. Tissue-to-background ratios (TiBR) were calculated by dividing the SUV_max_ of the organs by the SUV_mean_ of the background (gluteal muscle).

Three physicians with long-time experience in PSMA-targeted PET/CT (S.E., F.K., and F.R.) visually identified suspicious lesions taking into account all four imaging time points (1 h, 24 h, 48 h, and 72 h p.i.). Lesions that were visually considered as suggestive for prostate cancer were analyzed by measuring SUV_max_ and by calculating tumor-to-background ratios (TBR), defined as SUV_max_ divided by SUV_mean,_ of the background (gluteal muscle).

### Radiation dosimetry

Mean absorbed radiation doses were estimated using the QDOSE program package (ABX-CRO, Dresden, Germany) considering the following organs as source organs: kidneys, liver, spleen, salivary glands (parotid gland and submandibular gland), and lacrimal gland. As a first step, volumetric co-registration of the different time points was performed by taking the first CT scan as reference. PET images, which were coupled to the CT images of the respective imaging session, were transformed according to the CT transformation matrix. Boundary VOIs which solely enclosed the source organs without interfering with the activity concentration of neighboring structures were manually drawn in the PET image that allowed the best organ delineation and then copied onto all other time-point scans. Manual adjustment of the boundary VOI for each time point was done when necessary, using the respective CT. Volume and activity estimation were performed within the boundary applying a fuzzy locally adaptive Bayesian (FLAB) segmentation algorithm for automatic volume delineation [24]. The next step comprised mono-exponential regression of the serial measured activities using weighted least squares method and estimation of both, the time integrated activities (TIA) and the time-integrated activity coefficients (TIAC) in the source regions. As an approximation, a linear increase from t = 0 to the first acquisition time point was assumed, and the integration method for the first time interval was based on the trapezoidal method. Between the first and the last time point, a trapezoidal integration was used, whereas the TIA from the last time-point to infinity was calculated analytically by applying the mono-exponential fitting curve. The respective TIACs (often termed residence time) of the source organs were calculated by normalizing the TIA to the amount of activity administered. The total body TIA was calculated approximately using the total FOV volume of interest and the injected activity. As a further approximation, constant activity between t = 0 and the first acquisition time point was assumed, and the integration method for the first-time interval was based on the trapezoidal method. Between the first time point and infinity, the TIA was calculated analytically by using the mono-exponential fitting curve. Estimations of absorbed organ dose and effective dose were performed by the IDAC 2.1 software which is implemented in QDOSE [25–27]. The IDAC reference man was applied for the kidneys, the spleen, the liver, the heart and the intestine, and the sphere model for the salivary and lacrimal glands. Patient-specific organ mass adjustment, which is available in QDOSE, was applied for the kidneys, the liver and the spleen. Respective organ masses were determined using the volume of each organ delineated from the CT images (PACS software, SECTRA, Linköping, Sweden) and the respective biological tissue density. Organ masses for the salivary glands were taken from International Commission on Radiological Protection (ICRP) publication 89 with 25 g estimated weight for the parotid and 12.5 g for the submandibular gland [28].

## Results

### Adverse events

The examination with [^89^Zr]Zr-PSMA-617 was not associated with any side effects in all 7 patients. No acute adverse events or other drug-related pharmacologic effects occurred. Monitored vital parameters remained unchanged. No patient complained of any subjective symptoms during examination and follow-up.

### Biodistribution

**Fig. 1:**
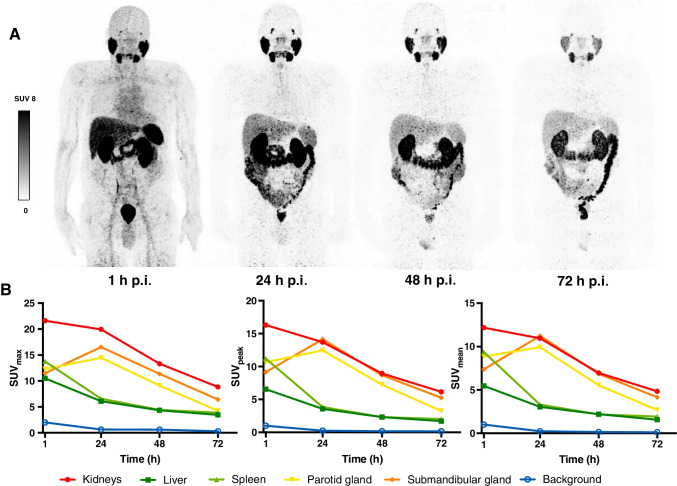
Representative example (patient no. 2) **A** Maximum intensity projections at 4 time points post injection (p.i.) of [^89^Zr]Zr-PSMA-617, **B** Respective SUV_max_, SUV_peak_ and SUV_mean_ kinetics in normal organs (kidneys, liver, spleen, parotid gland, submandibular gland) and background (gluteal muscle)

Intense physiological uptake was observed in the salivary and lacrimal glands, liver, spleen, kidneys, intestine, and urinary tract. Figure 1 demonstrates a representative example of [^89^Zr]Zr-PSMA-617 PET/CT at the predefined time points 1 h, 24 h, 48 h, and 72 h p.i., including the respective biodistribution data of selected organs assessed by SUV kinetics.

**Fig. 2:**
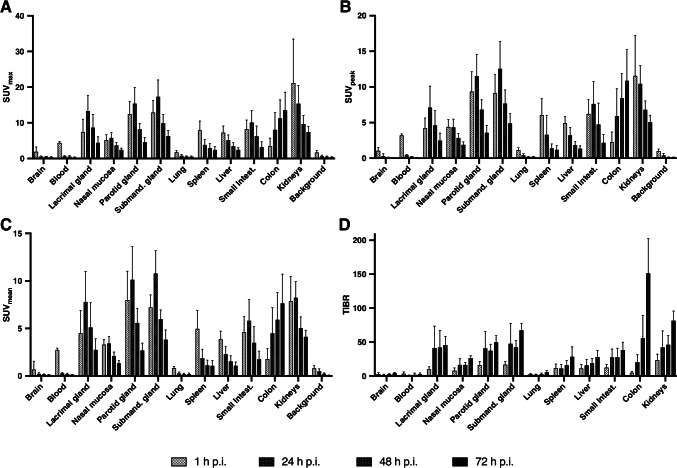
Biodistribution of [^89^Zr]Zr-PSMA-617 with descriptive statistics (mean ± standard deviation) of **A** SUV_max_, **B** SUV_peak_, and **C** SUV_mean_ and **D** tissue-to-background ratio (TiBR) in normal organs at 1 h, 24 h, 48 h, and 72 h p.i.

Detailed descriptive statistics of tracer distribution of all patients, including SUV_max_, SUV_peak_, SUV_mean_ and TiBR (tissue-to-background-ratio) of various organs, are presented in Fig. 2. The highest average SUV_max_ at 1 h p.i. was observed for the kidneys with 21.15 ± 12.31, consequently decreasing to 7.43 ± 1.56 at 72 h p.i.. For salivary glands and lacrimal glands, the maximum SUV_max_ was at 24 h p.i., with the highest value for submandibular gland (17.30 ± 4.69 at 24 h p.i., decreasing to 6.29 ± 1.61 at 72 h p.i.). Only colonic tracer uptake increased continuously (from 3.50 ± 2.21 at 1 h p.i. to 13.57 ± 5.00 at 72 h p.i.). TiBR increased between 1 and 72 h p.i. for the salivary glands, nasal mucosa, liver, colon, and kidneys. The highest TiBR values (≥ 50) were observed in salivary glands, kidneys, and colon at 72 h p.i.

### Radiation dosimetry

The monoexponential curve-fitting parameters, the time-integrated activity coefficients (TIAC) for each source organ, and the respective estimated absorbed doses according to IDAC 2.1 are summarized in Table 2. Among the normal tissues, the parotid gland received the highest absorbed dose of [^89^Zr]Zr-PSMA-617 with 0.601 ± 0.185 mGy/MBq followed by the kidneys with 0.517 ± 0.125 mGy/MBq and the submandibular gland with 0.468 ± 0.136 mGy/MBq. These values resulted in an average effective dose of 0.0913 ± 0.0118 mSv/MBq. Thus, administration of 111 MBq of [^89^Zr]Zr-PSMA-617 (mean injected activity) induced a total effective dose of 10.1 mSv.

**Table 2:** Monoexponential curve-fitting parameters, time-integrated activity coefficients (TIAC), and mean absorbed dose estimate for [^89^Zr]Zr-PSMA-617 in selected organs. Results are presented as mean values ± standard deviation

Organ	A (% injected A_0_)	l (h^−1^)	TIAC (h^−1^)	Absorbed dose (mGy/MBq)
Kidneys	3.75 ± 1.24	0.022 ± 0.002	1.74 ± 0.44	0.517 ± 0.125
Liver	6.62 ± 1.39	0.049 ± 0.014	1.54 ± 0.49	0.158 ± 0.051
Spleen	1.25 ± 0.70	0.079 ± 0.034	0.21 ± 0.12	0.175 ± 0.068
Parotid gland	0.70 ± 0.20	0.031 ± 0.005	0.31 ± 0.10	0.601 ± 0.185
Submand. gland	0.27 ± 0.09	0.029 ± 0.004	0.13 ± 0.04	0.468 ± 0.185
Lacrimal gland	0.02 ± 0.01	0.030 ± 0.006	0.10 ± 0.004	0.156 ± 0.070

*A*, activity; *l*, rate constant; *TIAC*, time-integrated activity coefficient

### Clinical findings and tumor uptake

**Fig. 3:**
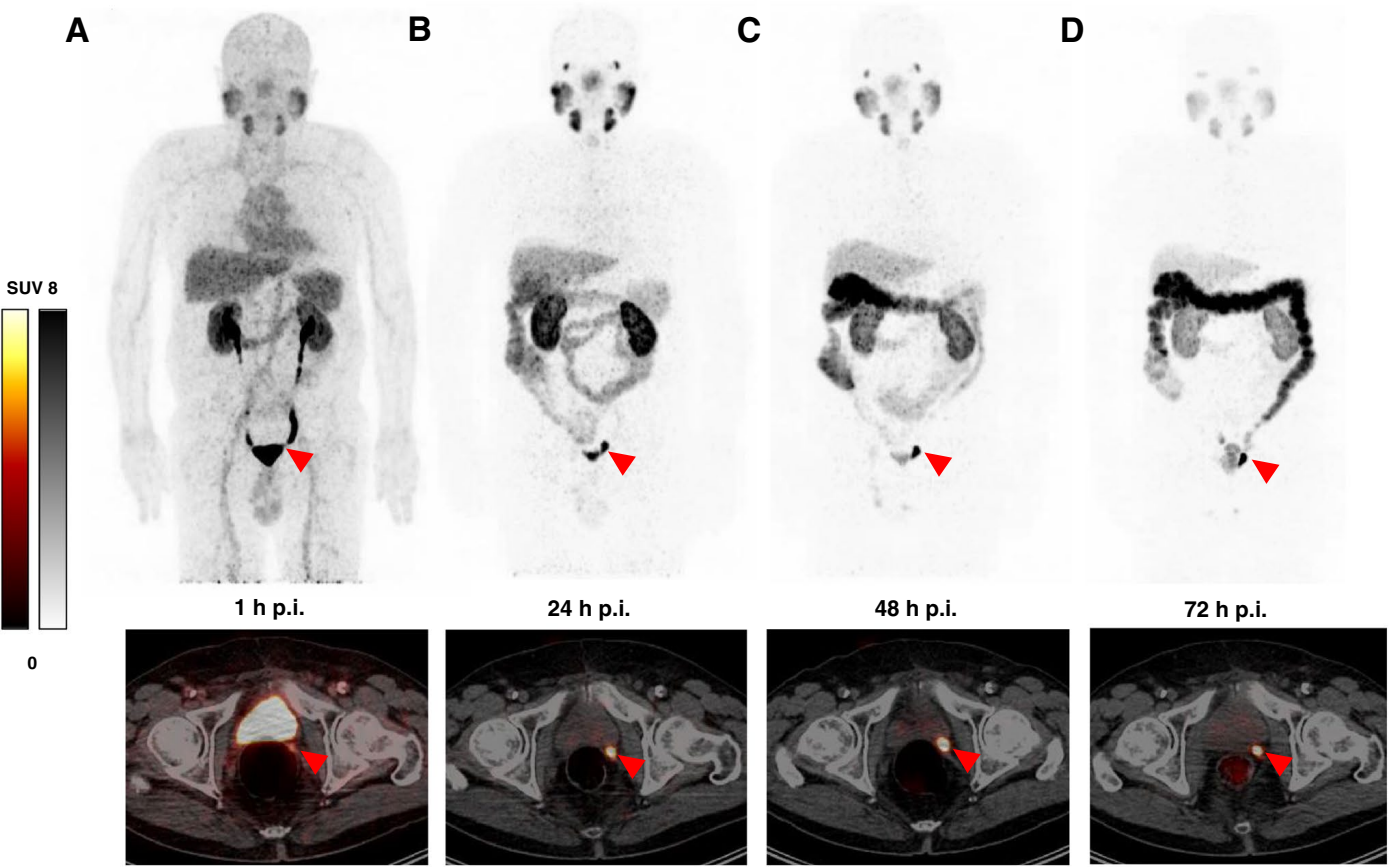
Maximum intensity projections (MIP) and transversal PET/CT slices of patient no. 6 (PSA 0.43 ng/ml) at 1 h (**A**), 24 h (**B**), 48 h (**C**), and 72 h (**D**) post injection (p.i.) of [^89^Zr]Zr-PSMA-617. Red arrows point to a suspicious focal uptake in the left seminal vesicle bed showing increased tracer uptake at 24 h p.i., 48 h p.i., and 72 h p.i. compared to 1 h p.i., therefore considered as local recurrence. SUV_max_ at 1 h / 24 h / 48 h and 72 h p.i.: 7.04 / 21.27 / 21.09 / 19.19

**Fig. 4:**
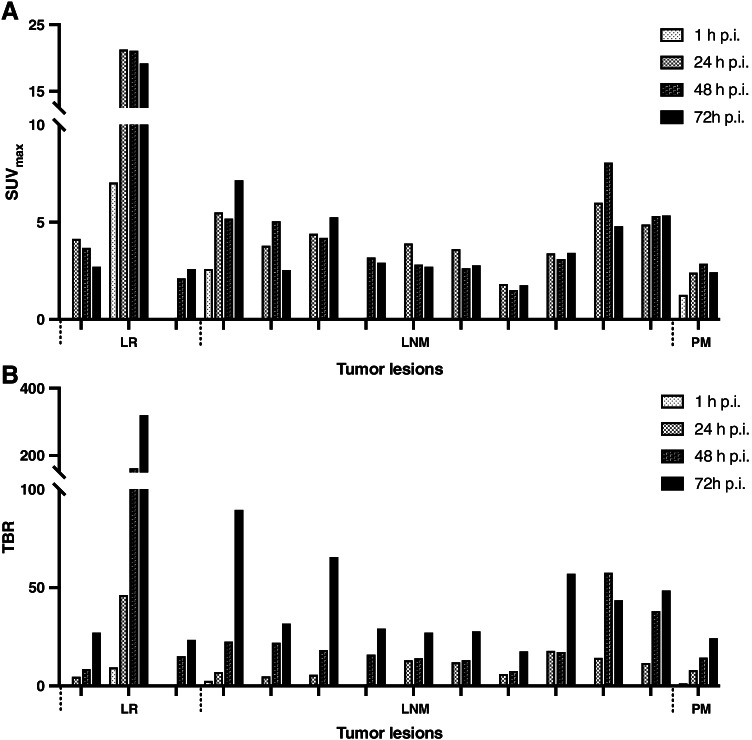
SUV_max_ (**A**) and tumor-to-background ratio (TBR) (**B**) of all tumor lesions at 1 h, 24 h, 48 h, and 72 h post injection (p.i.) of [^89^Zr]Zr-PSMA-617. LR, local recurrence; LNM, lymph node metastasis; PM, peritoneal metastasis

Among 7 patients with either negative (4 patients) or with undetermined findings on [^68^Ga]Ga-PSMA-11 PET/CT (3 patients), at least one lesion (range 1–5 per patient, in total *n* = 14) that was suggestive for prostate cancer was detected in 6 patients with [^89^Zr]Zr-PSMA-617 (Table 1). Out of all identified lesions (*n* = 14), 10 were lymph node metastases (in 4 patients), 3 were local recurrence (in 3 patients), and 1 was peritoneal metastasis. The majority of tumor lesions (*n* = 11) were not visible on PET/CT imaging at 1 h p.i., but were delineated at later time points (9 of them first visible at 24 h p.i., and 2 at 48 h p.i.). All tumor lesions were detectable at 48 h p.i. and 72 h p.i.. No additional tumor lesions were found at 72 h p.i.. Three tumor lesions (in 3 patients), which were identified at 1 h p.i., were also visible in [^68^Ga]Ga-PSMA-11 PET/CT. An exemplary tumor lesion and its uptake over time is shown in Fig. 3. A graphical representation of the SUV_max_ and TBR values of all lesions in all patients is provided in Fig. 4. In those lesions, which could be identified from early imaging (1 h p.i), SUV_max_ further increased substantially from 1 to 24 h p.i.. In late imaging (≥ 24 h p.i.), SUV_max_ plateaued in all lesions and TBR increased continuously over time.

**Fig. 5:**
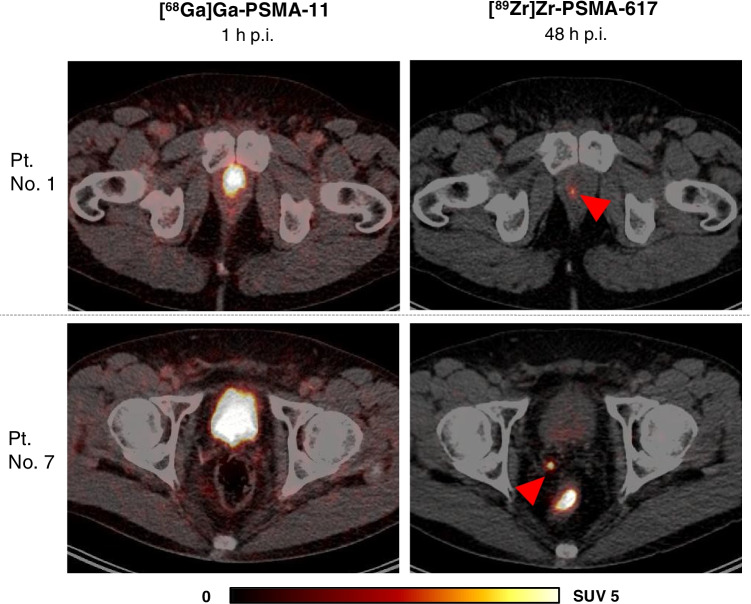
Transversal slices of [^89^Zr]Zr-PSMA-617 (right) and [^68^Ga]Ga-PSMA-11 (left) PET/CT of 2 patients (patient no. 1 and 7) with PSMA-positive lesions detected by [^89^Zr]Zr-PSMA-617 but unidentified by [^68^Ga]Ga-PSMA-11. Red arrows point to suspected focal uptake. Patient no. 1 (PSA 1.92 ng/ml) with focal uptake (SUV_max_ 3.68) in the prostate bed considered as local recurrence. Patient no. 7 (PSA 0.68 ng/ml) with focal uptake (SUV_max_ 8.06) in a pelvic lymph node considered as lymph node metastasis

**Fig. 6:**
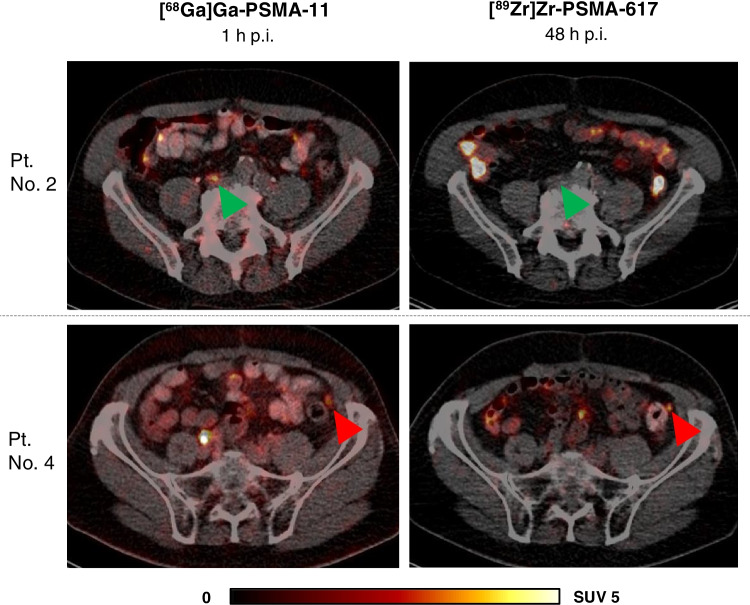
Transversal slices of [^89^Zr]Zr-PSMA-617 (right) and [^68^Ga]Ga-PSMA-11 (left) PET/CT of 2 patients (patient no. 2 and 4) with undetermined PSMA-positive findings on [^68^Ga]Ga-PSMA-11 PET/CT clarified by [^89^Zr]Zr-PSMA-617 PET/CT. Green arrows point to pelvic tracer uptake (SUV_max_ 4.47) on [^68^Ga]Ga-PSMA-11 PET/CT 1 h p.i. with no corresponding uptake on [^89^Zr]Zr-PSMA-617 PET/CT, considered as unspecific uptake. Red arrows point to faint tracer uptake (SUV_max_ 2.06) in a peritoneal lesion on [^68^Ga]Ga-PSMA-11 PET/CT 1 h p.i. with corresponding uptake (SUV_max_ 2.87) on [^89^Zr]Zr-PSMA-617 PET/CT 48 h p.i. considered as peritoneal metastasis

In 3 of 4 (75%) patients with previously negative [^68^Ga]Ga-PSMA-11 PET/CT, lesions that were visually considered as suggestive for prostate cancer were identified using [^89^Zr]Zr-PSMA-617. Two patients were found to have focal uptake in the prostate bed and one of these two and another patient revealed uptake in lymph nodes suggesting local recurrence and lymph node metastases, respectively (Table 1). Figure 5 exemplifies a local recurrence and a lymph node metastasis visible on [^89^Zr]Zr-PSMA-617 PET/CT at 48 h p.i., which could not be identified by [^68^Ga]Ga-PSMA-11 PET/CT. In 2 of 3 patients with undetermined findings in [^68^Ga]Ga-PSMA-11 PET/CT, the respective findings were confirmed by [^89^Zr]Zr-PSMA-617 PET/CT, whereas in 1 of 3 patients, no corresponding uptake was identified (Fig. 6). Furthermore, in 2 of these 3 patients, additional lesions were detected in [^89^Zr]Zr-PSMA-617 PET/CT, which were unidentified by [^68^Ga]Ga-PSMA-11 PET/CT (Table 1).

Subsequently, five patients received radiotherapy adjusted according to the results of [^89^Zr]Zr-PSMA-617-PET/CT and in the other two patients, ADT was initiated. There were minor (e.g., additional boost) and also major adjustments on radiation treatment planning (e.g., extension of the radiation field) by the findings on [^89^Zr]Zr-PSMA-617-PET/CT. In all 5 patients who received radiation therapy, a PSA decrease was achieved, in 4 patients by more than 70% and in 1 patient of 30%.

## Discussion

This is a pilot study presenting first clinical experience of [^89^Zr]Zr-PSMA-617 PET/CT including biodistribution, radiation dosimetry estimates, and analysis of tumor lesion imaging in patients with BCR of prostate cancer.

Comparing the biodistribution of [^89^Zr]Zr-PSMA-617 with that of [^68^Ga]Ga-PSMA-617 and [^68^Ga]Ga-PSMA-11, a similar distribution pattern was observed at 1 h p.i. with intense uptake of radiolabeled PSMA ligands in the salivary glands, lacrimal glands, kidneys, liver, spleen, and small intestine [29, 30]. In contrast to established ^68^Ga-labeled PSMA tracers, the relatively long half-life of ^89^Zr with T_1/2_ = 78.4 h allowed additional imaging at later time points (up to 72 h in our study). While radiotracer uptake in kidneys, liver, and spleen was continuously decreasing over time, it was highest in the salivary glands, lacrimal glands, and small intestine at 24 h p.i. and decreased thereafter. In addition, all of our patients showed increasing [^89^Zr]Zr-PSMA-617 radiotracer uptake in the colon over time. We suggest that tracer excretion is not only through the urinary system, but more significantly at later time points, also by the intestinal system with intense tracer accumulation in the colon. Comparable observations have also been reported in [^177^Lu]Lu-PSMA-617 scintigraphy for dosimetry measurements [31, 32]. In contrast to the normal organ uptake, radiotracer accumulation in tumor lesions increased until 24 h p.i. and then remained essentially stable up to 72 h p.i., which may be explained by the internalization process of the radioligand/PSMA complex and subsequent radiotracer trapping. Together with the continuing clearance from non-target tissues, this obviously leads to significantly higher TBR values, which may result in an improved detection of tumor lesions (vide infra).

The estimated overall effective dose of [^89^Zr]Zr-PSMA-617 PET for the administration of 111 MBq was 10.1 mSv (0.0913 ± 0.0118 mSv/MBq) and therefore about 2–3 times higher than PET with [^68^Ga]Ga-PSMA-617 or [^68^Ga]Ga-PSMA-11 with a respective diagnostic activity of 200 MBq [29, 30]. The parotid glands and kidneys were the organs receiving the highest absorbed doses analogue to [^68^Ga]Ga-PSMA-617 and [^68^Ga]Ga-PSMA-11, but about 3–4 times higher in absolute numbers. These results could be expected and were attributed to the longer half-life of ^89^Zr compared to ^68^Ga. However, our radiation dose estimates are lower than the reported absorbed doses of [^89^Zr]Zr-PSMA-DFO, another most recently introduced ^89^Zr-labeled PSMA ligand (effective dose 0.15 ± 0.04 mSv/MBq) [33]. Not surprisingly, the radiation exposure of [^89^Zr]Zr-PSMA-617 PET/CT did not induce any of the side effects which have been reported for targeted radiotherapy of metastatic prostate cancer with PSMA-ligands, e.g., dry mouth and mucositis. Also, no other side effects were observed. From our pilot experience, [^89^Zr]Zr-PSMA-617 PET/CT can be considered safe.

The most important clinical finding was that in 3 of 4 patients (75%) with negative [^68^Ga]Ga-PSMA-11 PET/CT, prostate cancer lesions (local recurrence or lymph node metastases) were identified by [^89^Zr]Zr-PSMA-617 PET/CT. Furthermore, in 3 patients with undetermined findings on [^68^Ga]Ga-PSMA-11 PET/CT, [^89^Zr]Zr-PSMA-617 PET/CT was able to clarify the results and in 2 of these patients to identify additional tumor lesions. Despite the high diagnostic performance of PET/CT with ^68^Ga-labeled PSMA radiotracers in patients with BCR of prostate cancer, a considerable proportion of negative findings has also been reported, particularly in patients with low PSA levels [11–14]. Here, [^89^Zr]Zr-PSMA-617 PET/CT seems to be a useful complementary examination. The increased radiotracer uptake in tumor lesions at late imaging time points together with decreasing activity in the blood pool, in normal tissues, e.g., the urinary tract and particularly in the bladder, allowed the detection of lesions that were not visible at early imaging time points. Our observations are in accordance with the study of Dietlein et al. who reported detection of prostate cancer lesions by [^89^Zr]Zr-PSMA-DFO PET/CT (at 2–3 days p.i.) in 8/14 (57%) patients with initially negative PSMA-targeted PET/CT ([^68^Ga]Ga-PSMA-11 or [^18^F]-JK-PSMA-7) [33]. The more precise localization of tumor lesions in patients with BCR by ^89^Zr-labeled PSMA PET/CT is likely to have consequences for therapy management, e.g., in our cohort of patients, radiotherapy treatment was adjusted for all 5 patients who received subsequent salvage radiotherapy (either minor adjustments as, e.g., additional boost or major adjustments as e.g. extension of radiation field). In this respect, PET/CT with ^89^Zr-labeled PSMA tracers may contribute to an improved and individualized therapy concept for patients with BCR.

Whether [^18^F]F-PSMA-1007 PET/CT would have detected or clarified the lesions of our cohort due to hepatobiliary excretion with almost complete absence of activity in the bladder and the moderately longer half-life compared to ^68^Ga remains speculative and requires future studies. Late PET/CT imaging with ^18^F-labeled PSMA tracers was reported and shown to result in increased tumor-to-background ratios at 3 h after tracer injection when compared to image acquisition 1 h p.i. [16]. However, due to the half-life of ^18^F of 110 min, respective PET/CT imaging is restricted to the day of tracer injection.

From our preliminary experience, imaging at 48 h p.i. seems to be the optimal time point for [^89^Zr]Zr-PSMA-617 PET/CT acquisition in patients with BCR. At 1 h and 24 h p.i., not all lesions could be detected, whereas at 48 h p.i. and 72 h p.i., all lesions were well delineated with comparable radiotracer uptake. In addition, compared to 72 h p.i. imaging, there was substantially less uptake of the radiotracer in the sigmoid colon and rectum at 48 h p.i., therefore allowing better identification of small regional lymph nodes. Furthermore, imaging at 48 h p.i. compared to 72 h p.i. is less exhausting for the patient due to shorter acquisition time. However, the most appropriate imaging time point needs further evaluation in larger patient cohorts.

Despite these promising results of [^89^Zr]Zr-PSMA-617 PET/CT in the detection of lesions and the potential advantage over established [^68^Ga]Ga-PSMA-11 PET/CT imaging, the increased radiation exposure of about 10 mSv overall effective dose should be considered and further studies are recommended in larger patient cohorts to identify the optimal administered activity to further minimize the radiation dose while still obtaining high-quality PET images at later time points. [^89^Zr]Zr-PSMA-617 PET/CT should thus be considered primarily when [^68^Ga]Ga-PSMA-11 PET/CT do not provide a correlate for a BCR or undetermined findings arise.

[^89^Zr]Zr-PSMA-617 PET/CT may have a paramount impact in the radioligand therapeutic field by enabling pre-therapeutic biokinetic studies. Due to the long half-life, a pre-therapeutic estimation of the resulting absorbed doses of PSMA-targeted radioligand therapy with [^177^Lu]Lu-PSMA-617 might be possible by [^89^Zr]Zr-PSMA-617 PET/CT since delayed imaging allows individual determination of the biological half-life of each tumor lesion and of the organs-at-risk. This may allow more personalized treatment regimens with administration of individually calculated activities and also prediction of toxicity. Furthermore, its half-life allows the possibility of centralized production and shipment to more distant imaging sites, which do not have the possibility for in-house production of [^68^Ga]Ga-PSMA-11. This study may serve as a rational starting point for further studies, ideally in a prospective setting, to confirm and extend our findings.

The results reported herein should be considered in the light of some limitations. The data are based on a retrospective monocenter study with a limited number of patients. Imaging was performed only at 1 h, 24 h, 48 h, and 72 h p.i.. The lack of additional imaging, particularly between 1 and 24 h p.i. and after 72 h, may affect dose estimates. In addition, excretion was not measured quantitatively. Furthermore, imaging findings were only confirmed by clinical course and biochemical follow-up and not by histology. In addition, no standardized companion imaging with MRI was performed. Also, no follow-up [^89^Zr]Zr-PSMA-617 PET/CT post-treatment is yet available.

## Conclusion

[^89^Zr]Zr-PSMA-617 PET/CT imaging is a promising new diagnostic tool with acceptable radiation exposure for patients with prostate cancer especially when [^68^Ga]Ga-PSMA-11 PET/CT imaging fails detecting recurrent disease. The long half-life of ^89^Zr (T_1/2_ = 78.4 h) enables late time point imaging (up to 72 h in our study) with increased tracer uptake in tumor lesions and higher tumor-to-background ratios allowing identification of lesions non-visible on [^68^Ga]Ga-PSMA-11 PET/CT imaging. Further studies, ideally in a prospective setting with larger patient cohorts, are recommended to confirm this observation of paramount impact for management of biochemical recurrence in prostate cancer.

## Data Availability

The datasets used and analyzed during the current study are available from the corresponding author on reasonable request. A case report of one patient included in this study has been previously published [34].

## Abbreviations

ADT: Androgen-deprivation therapy
BCR: Biochemical recurrence
cGMP: Current good manufacturing practice
CT: Computed tomography
DT: Doubling time
FLAB: Fuzzy locally adaptive Bayesian
FWHM: Full width half maximum
ID: Initial diagnosis
LA: Lymphadenectomy
OSEM: Ordered-subset expectation maximization
PET: Positron emission tomography
PSA: Prostate-specific antigen
PSMA: Prostate-specific membrane antigen
RP: Radical prostatectomy
RT: Radiation therapy
SUV: Standard uptake value
TBR: Tumor-to-background ratio
TIA: Time integrated activity
TIAC: Time-integrated activity coefficient
TiBR: Tissue-to-background ratio
VOI: Volume of interest
